# Exploring Spatial Differences Between 2 US Firearm Mortality Data Sets in 2017

**DOI:** 10.5888/pcd17.200096

**Published:** 2020-10-22

**Authors:** Meghan K. Herring, Cassandra A. Kersten

**Affiliations:** 1 Department of Epidemiology, Rollins School of Public Health, Emory University, Atlanta, Georgia

**Figure:**
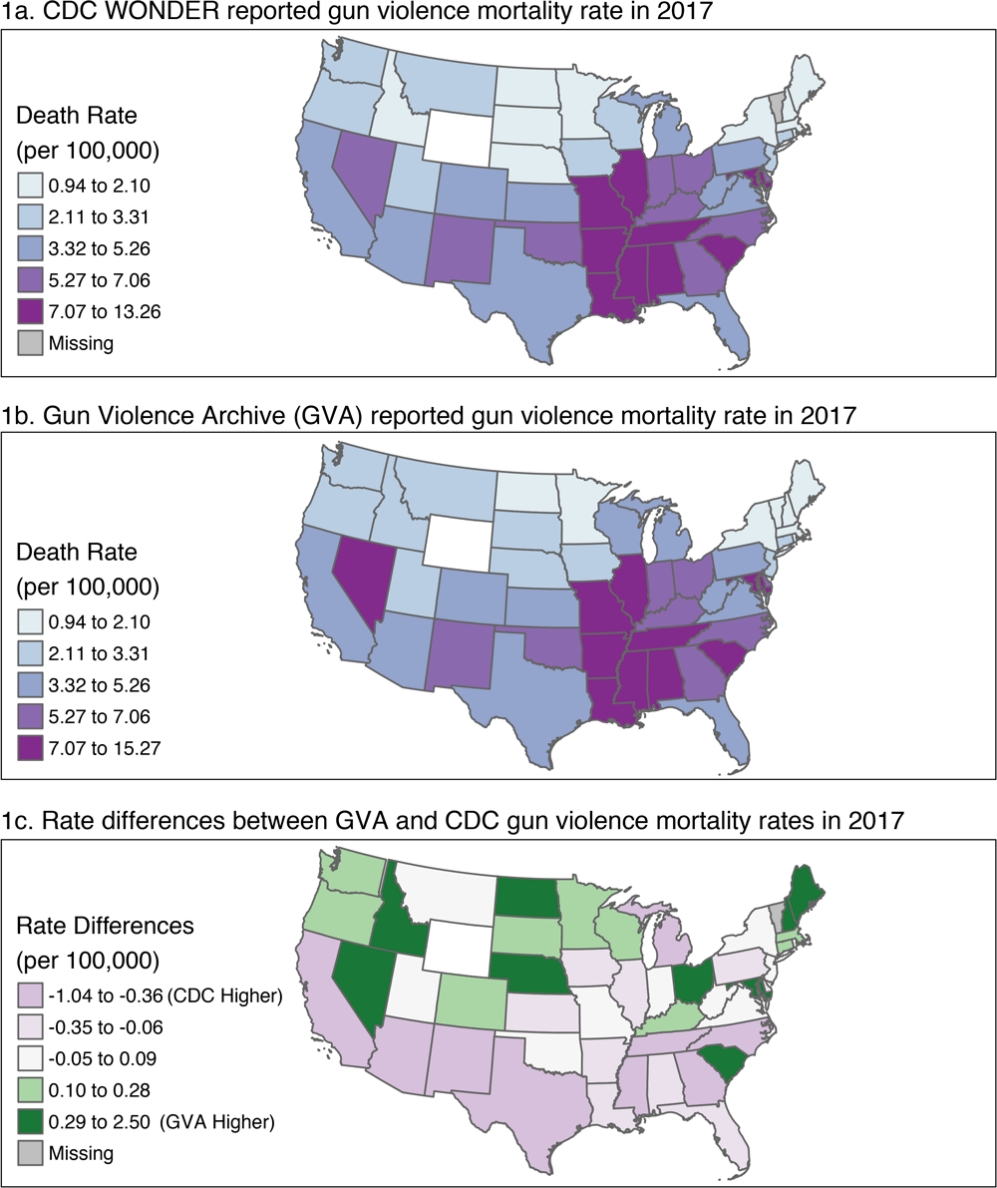
Spatial distribution of unadjusted death rate per 100,000 population of deaths from gun violence across the contiguous United States, by state, in 2017 in 2 data sets: the Centers for Disease Control and Prevention (CDC) Wide-ranging OnLine Data for Epidemiologic Research (WONDER) database (1) (panel 1a) and the Gun Violence Archive (GVA) (2) (panel 1b). Panel 1c shows the rate differences between the 2 data sets. Population data are from the US Census Bureau (3).

**Figure:**
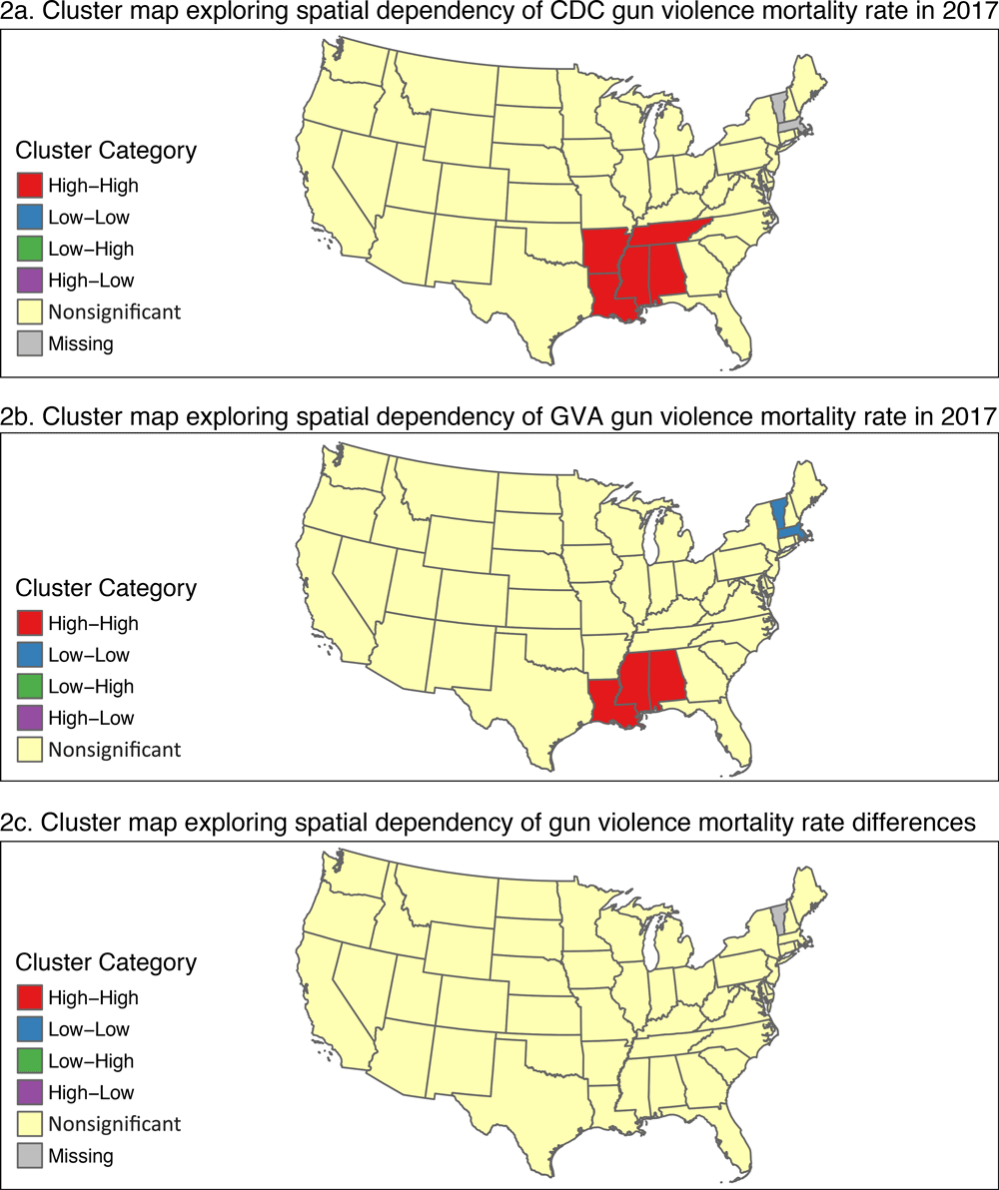
Cluster maps of the spatial dependency of gun violence mortality rates across the contiguous United States, by state, in 2017 in 2 data sets: the Centers for Disease Control and Prevention (CDC) Wide-ranging OnLine Data for Epidemiologic Research (WONDER) database (1) (panel 2a) and the Gun Violence Archive (GVA) (2) (panel 2b). Panel 2c shows the rate differences between the 2 data sets. Population data are from the US Census Bureau (3). Local Moran’s I (4) was used to calculate mortality rate differences between the states. States with missing data were suppressed because of small numbers in CDC WONDER.

## Background

Firearm-related mortality and its relation to public health has been a controversial topic in the United States for many years. The most commonly used source for research on gun-related deaths is the Centers for Disease Control and Prevention (CDC) Wide-ranging OnLine Data for Epidemiologic Research (WONDER) database, which compiles data from death certificates (1). However, there are several other published sources of gun violence data, including the Gun Violence Archive (GVA), which collects and compiles data from public news media sources (2). Although CDC WONDER is the most commonly used source for data on gun violence, concerns have been voiced around the validity of cause-of-death reporting on death certificates (5,6). We sought to compare deaths reported in CDC WONDER with deaths captured via media sources to compare the 2 mechanisms and evaluate their benefits and drawbacks. Through this project, we analyzed spatial similarities in 2017 between the CDC WONDER and GVA data sets for the contiguous United States. We compared the mortality rates in each state, the differences in the rates, and looked for evidence of spatial autocorrelation between the 2 data sets.

## Data Sources and Map Logistics

These data came from 3 different sources and excluded deaths attributable to self-harm or war-related violence. The first was CDC WONDER detailed mortality data from 2017 for External Causes of Death attributable to firearms (*International Classification of Diseases, 10th Revision* [ICD-10] codes X93, X94, X95, Y22, Y23, Y24, Y35.0, W32, W33, and W24) (1). For comparison, we used the GVA, an independent data collection and research group that collects data on gun violence deaths and injuries from law enforcement, media, and commercial sources with the goal of providing near real-time gun violence data (2). The spatial information came from the US Census Bureau (3).

We started by adding the spatial information provided by the US Census Bureau to each of the gun violence data sets. Within each data set, we then aggregated data to the state level in the contiguous United States to eliminate suppression of results by CDC WONDER because of small numbers. We completed a descriptive analysis by creating a disease map for each source of the mortality rate per 100,000 people split into quintiles based on the CDC data set values. We then ran a Global Moran’s I (7) test on each to evaluate spatial autocorrelation across the United States. Global Moran’s I tests the null hypothesis of random distribution against hypotheses that the presence of a gun violence death in 1 state is dependent on such presence in neighboring states (7). After finding evidence of global spatial autocorrelation, we ran a Local Moran’s I test (4) on each source using their rates to explore spatial autocorrelation at the local level. The Local Moran’s I test breaks down the Global Moran’s I and allows viewers to understand the contribution of each observation to identify states existing as outliers (4).

After conducting individual analyses, we then compared the differences in the rates between the 2 data sources. We mapped the rate differences where a positive rate difference indicated higher reporting from GVA and a negative rate difference indicated lower reporting by GVA when compared with CDC data. Using these differences, we then completed a spatial clustering analysis at the global and local level by using the Global Moran’s I test (7) and the Local Moran’s I test (4).

## Highlights

Despite both data sets separately showing significant spatial clustering, no significant clustering of the rate differences between the 2 data sets was apparent. Small differences in reported rates were observed and did appear to vary spatially; however, they were not significantly clustered. These reported rates may have varied slightly because of differences in mode of collection, with some states having more deaths related to gun violence detected by media reports and others having more cases detected through death certificates. However, these differences were very small and were not associated with spatial autocorrelation of rate differences. As such, these minor differences in death rates do not appear to be affected by nearby states, indicating a lack of systematic error by region that would affect the accuracy of either data set. This indicates that while these 2 data sets are comparable for conducting analyses across the continental United States, these minor rate differences may cause effects when conducting analyses on a state-to-state or county-level scale.

## Action

These findings indicate that either data set could be used for future projects looking at firearm mortality across the continental United States because of a lack of significant spatial clustering and only minor differences in the death rates between data sets. Each data set comes with its own unique challenges and benefits. The CDC WONDER data set is publicly available, has been collected and used for many years, and provides more reliable demographic data than GVA (1). However, CDC data may be difficult to use below the state level because of suppression of low numbers of events. The GVA data set provides more specific details on events, including precise latitude and longitude information, detailed data on the victims, and links to news articles with contextual information (2). However, these data must be requested for use and may include reporting biases from news sources that would be minimized in death certificate data. These results offer some flexibility to future researchers, as election for use between these 2 data sets can strictly depend on the research question of interest. One additional consideration for this type of research is the potential that both data sets are skewed in the same direction because they only capture reported deaths, though this may be minimized by capturing deaths through different mechanisms. Further research should be conducted on this topic to understand how reported death counts obtained through death certificates and news sources differ from actual deaths.
